# Setting – based prevalence and correlates of central obesity: findings from a cross-sectional study among formal sector employees in Dodoma City, Central Tanzania

**DOI:** 10.1186/s12889-020-10142-4

**Published:** 2021-01-07

**Authors:** Mariam John Munyogwa, Kaloli Sayi Ntalima, Secilia Ng’weshemi Kapalata

**Affiliations:** Department of Public Health, College of Health Sciences, University of Dodoma, Dodoma, Tanzania

**Keywords:** Central obesity, Waist circumference, Formal sector employees, Dodoma city, Central Tanzania

## Abstract

**Background:**

Obesity at the workplace has been associated with symptoms of lower self-esteem, increased individual and employer healthcare costs, increased absenteeism and presenteeism and reduced productivity. Therefore, this study was designed to study the prevalence and correlates of central obesity among formal sector employees in Dodoma City.

**Methods:**

Study design was a cross-sectional survey conducted from March to June, 2019. Participants were employees from formal sector employment defined as those paid regular monthly wage and with either a secured permanent or temporary contract. Simple random sampling was used to select four out of fifteen large buildings hosting various establishments. Respondents were obtained conveniently and interviewed face to face. Central obesity was defined as a waist circumference greater than 102 cm for males and greater than 88 cm for females. Chi-square test was conducted to assess the differences among the groups. Simple and multiple logistic regression models were fitted to identify the correlates of central obesity.

**Results:**

A total of 392 respondents (98% response rate) agreed and participated in the study. The overall prevalence of central obesity was found to be 41.8% (164/392). The prevalence of central obesity was significantly higher among females (67.4% *p* < 0.001), respondents aged ≥51 years (60%, p = < 0.001), administrators (55.1% p = < 0.05), respondents with salary of > 1,000,000 Tanzanian Shilling (TSh.) per month (54.4%, p = < 0.05), respondents who eat homemade meals at the workplace (64.2%, p = < 0.05) and respondents with hypertension (62.5%, p = < 0.05). Correlates of central obesity were found to be female sex (AOR = 9.53; 95% CI: 5.49, 16.78), increased age, eating homemade meals at the workplace (AOR = 2.32; 95% CI: 1.04, 4.19) and hypertension (AOR = 3.15; 95% CI: 1.41, 6.91).

**Conclusions:**

The present study revealed high prevalence of central obesity among formal sector employees in Dodoma City. Scholars and stakeholders are urged to generate more evidences and design appropriate interventions to curb the situation.

**Supplementary Information:**

The online version contains supplementary material available at 10.1186/s12889-020-10142-4.

## Background

Overweight and obesity are increasing worldwide [1–4] and the prevalence of obesity has almost doubled since 1980s [5, 6]. According to the World Health Organization (WHO) report of 2015, more than 1.9 billion adults (18 years and older) were overweight and among them, over 650 million were obese [5]. The root causes of obesity are complex, including genetics, socio-economical, cultural, environmental, emotional, behavioral and lifestyle factors.

Obesity is a significant health alarm because it predisposes individuals to several co-morbidities, including hypertension, dyslipidemia, coronary heart disease, type 2 diabetes, stroke, cancer and osteoarthritis [5, 7–9]. These co-morbidities shorten life expectancy while impairing quality of life [10–12]. At the workplace, obesity has also been associated with symptoms of lower self-esteem, increased individual and employer healthcare costs, increased absenteeism and presenteeism at work and reduced employee productivity [13–15]. Employees spend more than 50% of their time at the workplace [16] therefore workplace may play a major role in predisposing people to obesogenic environment including physical inactivity and unhealthy diet [16, 17].

In Tanzania, previous scholars have reported much information on generalized obesity [18–26] among the general population [21, 22, 24, 26, 27]. Nevertheless, previous studies have demonstrated that central obesity is the best predictor of cardiovascular risk factors particularly type 2 diabetes mellitus and hypertension rather than generalized obesity [28, 29]. Recently studies have demonstrated that, Tanzania like other developing countries is also experiencing a higher burden of cardiovascular risk factors particularly diabetes and hypertension [5, 24]. Furthermore, information on obesity that addresses specific occupational sector as well as central obesity in Tanzania is limited. Settings-based information on overweight and obesity are also recommended to help the stakeholders to plan for more appropriate interventions which are likely to be less costly and more effective as they reach individuals into their usual environment [5]. Therefore, the current study aimed to determine the prevalence and correlates of central obesity among employees from the formal sector in Dodoma City, central Tanzania. Findings of this study will provide insight into the obesity situation among the formal sector employees population in Dodoma City. This will guide scholars and other stakeholders to plan for future research and appropriate interventions toward this group. Further, the use of waist circumference to assess obesity will add to the existing limited literature on central obesity in the country and help to identify people at increased risk of cardiovascular diseases.

## Methods

### Study area

This study was conducted in Dodoma City, central Tanzania. Dodoma City is the capital of Dodoma Region and national capital of Tanzania, with an estimated population of 459,350 [30]. Dodoma Region is found at the centre and semi-arid area of Tanzania mainland. Within Dodoma City, the employment sector forms the major occupation for most of the residences. Currently, most of the government offices and services are found in Dodoma. This happened after the government official announcement of 2018 which required government establishments to shift from Dar es Salaam to Dodoma Region. This government restructuring had also resulted into shifting of many other national and international establishments from elsewhere countrywide to Dodoma Region. Most of these establishments are located in Dodoma City and currently most of the government and other services can be acquired within the city.

### Study design and study population

This study was a quantitative analytical cross-sectional survey. The survey was conducted from March to June, 2019. The study participants were employees from formal sector employment. Formal sector employees were defined as those receiving a regular monthly wage and have either secured a permanent or temporary contract. The employees were classified into three categories namely: administrative, technical and supportive staffs. Administrative staffs included those with administrative roles such as legislators, supervisors and managers; technical staffs included those who perform the organization’s specialized professional, scientific and technical activities and supportive staffs included those who support organization’s general activities such clerks, drivers and receptionists etc. Inclusion criteria were all employees who were employed for more than 1 month in the current office.

### Sample size and sampling procedures

Sample size was calculated by using the formula $n={z}^2p\frac{1-p}{e^2}$ whereby: n = sample size, z = 1.96, *p* = 31.35% [27] and e = 5%. With assistance from the office of Dodoma District Commissioner, we identified a total of fifteen large buildings which contained different establishments within the city. Simple random sampling was then used to select four buildings. Respondents were selected by using convenient sampling method. A total of 400 eligible participants were approached for the study.

### Data collection and study procedure

Participants’ information was obtained through face to face interviews using a structured pre-tested questionnaire by self reporting. The questionnaire was developed by the researchers after comprehensive literature review to meet the objectives of this study (see Additional file 1). Lifestyle behaviors were assessed based on past 7 days prior to interview. Waist circumference (WC) was measured using the WHO protocol for measuring waist and hip circumference. WC was taken approximately at the midpoint between the lower margin of the last palpable rib and the top of the iliac crest. Abdominal obesity was defined as a WC greater than 102 cm for males and greater than 88 cm for females [31]. Blood pressure was measured by using a sphygmomanometer and recorded in millimeters of mercury (mmHg). Measurement was taken while the patient was in sitting position. Hypertension was defined as systolic blood pressure of ≥140 and/or diastolic blood pressure of ≥90 mmHg or currently using hypertensive medication [32]. Anthropometric measurements and blood pressure were measured by researchers who were the nutritionist and medical doctor respectively. Both researchers underwent refresher training for taking the measurements prior to data collection.

### Data analysis

Data were analyzed using the Statistical Package for Social Sciences (SPSS) Version 22. Frequencies and percentages were used to describe the population characteristics. Comparison between groups was done by using chi-square test. Simple logistic regression models were fitted to evaluate each independent variable for its unadjusted association with abdominal obesity. All independent variables with *p*-value less than or equal to 0.25 in simple logistic regression models were included in the multiple logistic regression model [27] to evaluate the correlates of central obesity among employees. All probabilities were two-tailed and independent variables with *p* values < 0.05 were regarded as significant.

## Results

### General characteristics of the study respondents

Data analysis was done by using SPSS version 22. A total of 392 (98% response rate) respondents were involved in this study. Two hundred and fifty four (64.2%) were males and 138 (35.8%) were females. A majority 152 (38.8%) of the respondents were aged 31–40 years. The number of respondents from the public and private employment sector were 256 (65.3%) and 136 (34.7%) respectively. Regarding the level of education, most of the respondents had completed a bachelor degree or higher 247 (63.0%). A majority of respondents were employed as technical staff (184, 46.6%) and few were administrators 69 (17.6%). Most of the respondents 175 (44.6%) earn a monthly salary ranging from TSh. 400,000/= to 1,000,000/=. Regarding transport to and from the workplace, the majority 345 (88%) of the respondents were using either motor vehicle or motorcycle. Most of the respondents 350 (89.3%) were eating meals from food vendors at the workplace. About 151(38.5%) of the respondents reported alcohol consumption and only 6(1.5%) reported smoking. Ten percent of the respondents had hypertension. About 164 (41.8%) of the respondents had central obesity (Table 1).

**Table 1:** General characteristics of the study respondents (*N* = 392)

Respondents’ general characteristics	n	%
**Sex**		
Male	254	64.2
Female	138	35.8
**Age**		
≤ 30	102	26.0
31–40	152	38.8
41–50	93	23.7
≥ 51	45	11.5
**Employment sector**		
Public	256	65.3
Private	136	34.7
**Educational status**		
Non Bachelor degree	145	37.0
Bachelor Degree and higher	247	63.0
**Type of occupation**		
Supportive staff^a^	139	35.5
Professional/technical staff^b^	184	46.9
Administrative staff^c^	69	17.6
**Monthly salary**		
< 400,000	103	26.3
400,000 –1,000,000	175	44.6
> 1,000,000	114	29.1
**Means of transport to and from workplace**		
Walking/ bicycle	47	12.0
Motor vehicle	345	88.0
**Source of meals at workplace**		
Food vendors	350	89.3
Homemade	42	10.7
**Currently drinking alcohol**		
No	241	61.5
Yes	151	38.5
**Currently smoking**		
No	386	98.5
Yes	6	1.5
**Hypertension**		
No	352	89.2
Yes	40	10.2
**Central obesity**		
No	228	58.2
Yes	164	41.2

^a^support office activities: secretary, driver, attendants etc

^b^perform the office technical activities, engineer, teacher, accountant etc

^c^perform lead administrative roles: legislators, managers, office supervisors, head of department etc

### Prevalence of central obesity by different characteristics of the respondents

The results of chi-square test shows that, prevalence of central obesity was statistically significant different by sex, age, educational status, type of occupation, monthly salary, means of transportation to and from the workplace and hypertension status. It was noted that, the prevalence of central obesity was significantly higher among females (67.4%) compared to males (28.0%), *p* < 0.001. Regarding age, prevalence of central obesity was highest among the oldest age group (≥ 51 years) respondents and lowest among the youngest age group (≤ 30 years) respondents (60.0% versus 25.5%, *p* < 0.001). Respondents with a bachelor degree or higher had higher prevalence of central obesity than those with non-bachelor degree. With respect to type of occupation, prevalence was higher among the respondents with administrative posts (55.1%) than those working as professional (44.0%) and supportive (32.4%) staff, *p* < 0.05. Regarding monthly salary, prevalence of central obesity increased with increases in monthly salary (p < 0.05). With respect to source of meals at the workplace, the prevalence of central obesity was higher among respondents who eat homemade meals (64.3%) than those who eat meals from food vendors (39.1%). Regarding hypertension status, the prevalence of central obesity was significantly higher among respondents with hypertension compared to those with normal blood pressure (62.5% vs 39.5%, *p* < 0.05) (Table 2).

**Table 2:** Prevalence and correlates of central obesity by different selected characteristics of the respondents (*N* = 392)

Variable	Central Obesity (%)^**#**^	OR (95% CI)	***p***-value	AOR (95% CI	***p***-value
**Sex**			< 0.001		< 0.001
Male	71 (28.0)***	1		1	–
Female	93 (67.4)	5.33 (3.39,8.35)		9.53 (5.49, 16.78)	–
**Age**			< 0.001		0.018
≤ 30	26 (25.5)***	1		1	–
31–40	466 (43.4)	2.24 (1.29, 3.88)		2.05 (1.06, 3.96)	–
41–50	45 (48.4)	2.74 (1.49, 5.00)		2.58 (1.19, 5.57)	–
≥ 51	51 (60.0)	4.39 (2.08, 9.23)		4.15 (1.65, 10.42)	–
**Employment sector**			0.205	–	–
Public	113 (44.1)*	1		–	–
Private	51 (37.5)	0.76 (0.49, 1.16)		–	–
**Educational status**			0.14	–	–
Non bachelor degree	49 (33.8)**	1		–	–
Bachelor Degree and higher	115 (46.6)	1.71 (1.12, 2.61)		–	–
**Type of occupation**			0.006	–	–
Supportive staff	45 (32.4)**	1		–	–
Professional staff	81 (44.0)	1.64 (1.04, 2.60)		–	–
Administrative staff	38 (55.1)	2.56 (1.42, 4.63)		–	–
**Monthly salary (TSh)**			0.02	–	–
< 400,000	32 (31.1)**	1		–	–
400,000–1,000,000	70 (40.0)	1.48 (0.88,2.48)		–	–
> 1,000,000	62 (54.4)	2.65 (1.52, 4.62)		–	–
**Means of transport to and from workplace**			0.18	–	–
Walking/ bicycle	12 (25.5)**	1		–	–
Motor vehicle/ cycle	152 (44.1)	2.29 (1.15, 4.58)		–	–
**Source of meals at workplace**			0.002	–	0.041
Food vendors	137 (39.1)**	1		1	–
Homemade	27 (64.3)	2.79 (1.44, 5.45)		2.32 (1.04, 4.19)	–
**Current alcohol use**			0.151	–	–
No	94 (39.0)*	1		–	–
Yes	70 (46.4)	1.35 (0.89, 2.04)		–	–
**Hypertension**			0.006	–	0.005
No	139 (39.5)**	1		1	–
Yes	25 (62.5)	2.55 (1.30, 5.02)		3.12 (1.41, 6.91)	–

^#^Differences in prevalence was assessed by chi-square test and the p-value obtained were as follows; *** p = < 0.001, ** p = < 0.05,* p = ≥ 0.05

### Unadjusted odds ratio (OR) of central obesity among the respondents

The results of a simple logistic regression model show that sex, age, type of occupation, monthly salary, source of meals at workplace and hypertension status were significantly associated with central obesity (*p* < 0.05) (Table 2). Females were more than five times likely to have central obesity than males (OR = 5.33; 95% CI: 3.39, 8.35). Regarding age, the odds of having central obesity increased with age whereby oldest age group respondents (≥ 51 years) had a greater odds of having central obesity (OR = 4.39, 95%, CI: 2.08, 9.23) compared to respondents of lower age groups (*p* < 0.001). Respondents with administrative posts had greatest odds of having central obesity (OR = 2.56, 95% CI: 1.42, 4.63) than professional and supportive staffs. With respect to monthly salary, respondents with the highest monthly salary category (> TSh.1,000,000/= per month) had a greater odds of having central obesity (OR = 2.65, 95% CI: 1.52, 4.62) than respondents with lowest monthly salary category. The odds of having central obesity was almost three times higher among the respondents who eat homemade meals at the workplace (OR = 2.79, 95% CI: 1.44, 5.45) than those who were eating meals from food vendors. Respondents with hypertension had more than twice odds of having central obesity (OR = 2.55, 95% CI: 1.30, 5.02) than those with normal blood pressure.

Although the level of education was not statistically significantly associated with central obesity (*p* = 0.14), the respondents with a bachelor degree or higher had almost twice the odds of having central obesity (OR = 1.71; 95% CI: 1.12, 2.61) than respondents the lower educational level. Likewise, regarding means of transportation to and from workplace (*p* = 0.18), respondents who were using motor vehicles or motorcycles had more than twice odds of having central obesity (OR = 2.25, 95% CI: 1.15, 4.62) than respondents who were either walking or using a bicycle.

### Correlates of central obesity among the respondents

After adjusting for age and sex, the results of multiple logistic regression model analysis showed that employment sectors, type of occupation, monthly salary, educational level, means of transportation to and from the workplace and alcohol consumption were not significantly (p = ≥ 0.05) associated with central obesity. On the other hand, sex, age, source of meals at workplace and status of hypertension were significantly (*p* = < 0.05) associated with central obesity (Table 2). Females had more than nine odds of having central obesity than (AOR = 9.53; 95% CI: 5.49, 16.78). Regarding age, the odds of having central obesity increased with age and the oldest respondents (≥ 51 years) had the greatest odds of having central obesity (AOR = 4.15; 95% CI:1.65, 10.42) compared to youngest age groups (≤ 30 years). The odds of having central obesity were more than twice among respondents who were eating homemade meal at workplace (AOR = 2.32; 95% CI: 1.04, 4.19) compared to those who were eating meals from food vendors. Respondents with hypertension had more than three times odds of having central obesity (AOR = 3.15; 95% CI: 1.41, 6.91) compared to those with normal blood pressure.

## Discussion

This study found high prevalence of central obesity in the formal sector employment at Dodoma City. Similar to the current finding, high prevalence of central obesity among employees has been reported from previous study conducted in Tanzania [26]. On the other hand, the current prevalence is less than what was reported from the study conducted among primary school teachers in Dar es Salaam, Tanzania [7]. This difference could be due to the fact that Dar es Salaam has been the city for more many years (> 40 years) and it is the most urbanized area in Tanzania compared to Dodoma City. Being highly urbanized, people living in Dar es Salaam are more likely to be exposed to higher risks of unhealthy eating and sedentary lifestyles compared to people living in Dodoma City. Furthermore, unlike the previous study that involved only one occupation, the current study involved different occupations. People from different occupations are likely to have mixture of different behaviors and lifestyles including healthy and unhealthy ones which may influence their health status in different ways including central obesity. On the other hand, people from one occupation are more likely to share common behaviors and lifestyles which might influence their health in a similar way including central obesity [5]. Furthermore, the current prevalence is higher than the ones reported from previous studies conducted among the general population in Tanzania [24, 27] and elsewhere [1, 2]. The difference observed could be due to the differences in study population among these studies. This finding may suggest that formal sector employees might be at higher risk of central obesity compared to general population. However, the available evidences are still limited inorder to reach the conclusion hence we recommend more research in order to generate more evidences on the same.

Consistence with the previous studies [1, 2, 24, 33], this study found that females were more likely to have central obesity than males. Furthermore, the current finding demonstrates higher difference in obesity levels between the two genders. A previous study from Italy, has demonstrated that occupational risk factors for obesity differ by sex [17]. However, the current study could not find specific employment related factors to explain this finding therefore authors recommend future scholars to study more on this area. Nevertheless, previous studies conducted from general population have identified several factors associated with female obesity which could also partly explain the current finding including; physiological differences between males and females [34], low physical activities in women compared to men [33] and certain African traditions such as practice of resting and fattening girls in preparation for engagement and marriage, keeping indoor mothers during postpartum period [35] and men’s preferences for overweight women [36].

Many previous studies have reported that increased age is associated with risk of central obesity [1, 2, 4, 37–39]. Similarly, this study found that the likelihood of having central obesity increased significantly with age. The possible explanation for this could be reduced basal metabolism and physical activities as a result of ageing.

Overweight and obesity is a result of energy imbalance between calories consumed and calories expended [5]. It has been believed that ready to eat meals from restaurants, food vendors etc. contain high energy hence creates positive energy balance. Surprisingly in our study we found that, eating homemade meals at workplace was associated with higher chances of having central obesity among the respondents. However, lack of information on types of food and amount of food eaten could be a limitation to this finding [20]. A few previous studies have demonstrated that a typical African meal is characterized by higher energy composition [20, 40] while another study conducted among the employees at Kuwait Oil Company showed that meals from food vendors were healthier than the homemade meals [40]. Therefore, this finding gives an opportunity for other scholars to explore more on dietary behavior and meal composition among employees at workplace.

Furthermore, the current finding continue to support the already known phenomenon that central obesity is threat as it predisposes people to hypertension [5, 9, 41]. Intra-abdominal (visceral) fat deposition (central obesity) is linked to an increased risk for cardiovascular co-morbidities [42]. However, obesity and its associated non-communicable diseases can be prevented. Targeted interventions to promote healthier food intake and regular physical activity need to be emphasized to help protect the population against obesity and related co-morbidities. Such approaches are affordable and effective in prevention of obesity.

### Strengths and limitations

In the Tanzanian context this is the first study that involved employees from different occupations. It adds to the limited available evidences on central obesity. Also the study was conducted in Dodoma where there is limited information on obesity and is an urban environment that is rapidly transforming. The measurements for central obesity and hypertension were measured by qualified experts during the survey. However, this study had a number of limitations. The study was a cross sectional study, the study population were the employees from formal employment only sector hence the findings may not be generalized for all employees categories. Also, information on socio-demographic characteristics and other factors assessed were self-reported by the respondents which may be a source of information bias.

## Conclusions

The present study had revealed a high prevalence of central obesity among the employees from formal sector in Dodoma City. Furthermore, this study demonstrates that obesity among this population is higher compared to the general population. This suggests that employees from the formal sector employment may be at higher risk of central obesity and its related consequences. As such, it is important to identify those factors that contribute to the problem in order to design appropriate interventions. The results from this study allow us to have an understanding of the current central obesity situation at the formal sector employment in Dodoma City, central Tanzania. This serves as initial step as we plan further studies to collect more detailed evidences to guide future interventions both locally and nationally. Regardless of the study limitations, the current findings can be used to plan targeted interventions for obesity and assess the effectiveness in reducing the prevalence of central obesity and its corresponding health consequences in this population.

## Supplementary Information

**Additional file 1.** Questionnaire: Assessment on obesity and associated risk factors among formal sector employees in Dodoma City Council, Tanzania.

## Data Availability

The datasets used and/or analysed during the current study are available from the corresponding author on reasonable request.

## Abbreviations

WHO: World Health Organization
WC: Waist circumference
TSh: Tanzanian shilling
OR: Unadjusted odds ratio
AOR: Adjusted odds ratio
CI: Confidence interval
