# Supplementary material for: Setting – based prevalence and correlates of central obesity: findings from a cross-sectional study among formal sector employees in Dodoma City, Central Tanzania
Source: BMC Public Health. 2021 Jan 7;21:97. doi: 10.1186/s12889-020-10142-4 (PMC7792166; doi:10.1186/s12889-020-10142-4)
Supplement: Supplementary file 1 — Additional file 1. Questionnaire: Assessment on obesity and associated risk factors among formal sector employees in Dodoma City Council, Tanzania. [file 12889_2020_10142_MOESM1_ESM.docx]

**Appendix 1. Questionnaire**

**TITLE: ASSSESSMENT ON OBESITY AND ASSOCIATED RISK FACTORS AMONG FORMAL SECTOR EMPLOYEES IN DODOMA CITY CENTRAL TANZANIA.**

Questionnaire No………………………………….

Date………….…………………………………….

Name of interviewer ……………………………

**PART I: DEMOGRAPHIC INFORMATION**

1. Category of the institution
2. Public
3. Private
4. Sex……………………………………….
5. male
6. female
7. Date of birth ……………………….
8. What is your current age …………………
9. Marital status
10. Single
11. Married
12. Divorced/separated
13. Widow
14. Ward of residency ……………………….
15. Type of residence
    1. Urban
    2. Rural
16. Level of education
17. Primary level
18. Certificate holder
19. Degree holder
20. Masters, postgraduate and above
21. Type of occupation (Name) ………………………………
22. What is your basic salary per month? (Mention) ……………………….

**PART II: OTHER LIFESTYLE CHARACTERISTICS**

1. What means of transport do you use to travel to and from your work place?
2. Own/private car or motorcycle
3. Office/institution transport,
4. Public transport (commuter buses, motorcycle etc).
5. Bicycle
6. On foot
7. What is your source of food while at your work place?
8. I take from home
9. Eat from office restaurant
10. Buy from food vendors
11. In the last week, did you drink alcohol
12. Yes
13. No
14. How much do you drink per day?
15. <200mls
16. 200 - 600mls
17. >600mls
18. Do you currently smoke
19. Yes
20. No

**PART III: PHYSICAL MEASUREMENTS**

1. Weight (kg)
2. Height (cm)
3. Hip circumference (cm)
4. Waist circumference
5. Blood pressure
